# Assessment of Mesiobuccal Canals in Endodontically Treated Teeth in Indore Population by Cone Beam Computed Tomography: A Cross-Sectional Study

**DOI:** 10.7759/cureus.58265

**Published:** 2024-04-14

**Authors:** Triveni Bhargava, Nimesh Jain, Prashanthi Reddy, Neelam Vijaywargiya, Vanaja Reddy, Haritima Nigam

**Affiliations:** 1 Oral and Maxillofacial Surgery, Government College of Dentistry, Indore, IND; 2 Oral Medicine and Radiology, College of Dental Science and Hospital, Indore, IND; 3 Oral Medicine and Radiology, Government College of Dentistry, Indore, IND; 4 Conservative Dentistry and Endodontics, Government College of Dentistry, Indore, IND; 5 Oral Medicine and Radiology, Modern Dental College and Research Centre, Indore, IND; 6 Oral Medicine and Radiology, Pacific Dental College, Kanpur, IND

**Keywords:** cbct study, root canal treatment, periapical radiolucencies, mesiobuccal canal, cone beam computed tomography

## Abstract

Background: Owing to the complicated anatomical nature of maxillary molars, untreated root canals may directly affect the outcome of root canal therapy. Therefore, cone beam computed tomography (CBCT) scan is an important tool in the evaluation of root canal systems, particularly for the detection of the second mesiobuccal (MB2) canal in maxillary molars.

Aims and objectives: The current study was undertaken to detect and evaluate filled/unfilled MB2 canals in endodontically treated, asymptomatic maxillary molars, and its correlation with periapical pathology by utilizing cone beam computed tomography (CBCT).

Material and method: A retrospective study of 80 CBCTs of patients underwent scanning for various treatment modalities, with asymptomatic endodontically treated permanent maxillary first molars selected. Data collection occurred between January and June 2023. CBCT machine used was KODAK 9000 (Rochester, NY: Carestream Health) (Complementary Metal Oxide Semiconductor {CMOS} sensor, continuous mode and 12-28 sec scan time, 90-500 μm voxel size, and 5x3.5 cm field of view {FOV}). The axial images at mid-root level were used to assess the presence of the MB2 canal.

Result: The study included 39 (48.8%) right maxillary first molars and 41 (51.3%) left maxillary first molars. Overall, in 62 (77.5%) maxillary first molars, MB2 was missed by the practicing dentist, and in 13 (16.3%) maxillary first molars MB2 canal was not present. Of all the maxillary first molars with MB2 canal (n=67), 53 (79.1%) canals had a periapical infection, five (7.5%) showed widening of periodontal ligament space whereas nine (13.4%) had no abnormality.

Conclusion: MB2 canals were present in the majority of cases and most of the unfilled MB2 canals showed evidence of periapical radiolucencies and showed a direct impact on the prognosis.

## Introduction

Apical periodontitis is a bacterial colonization of necrotic root canals that causes inflammation of the dental periradicular tissues. The microbial colonization of necrotic pulp tissues causes primary apical periodontitis. Secondary apical periodontitis develops as a result of a prolonged infection of improperly treated root canals [1]. Endodontic treatment is a procedure used to relieve pain and preserve teeth that are diseased. The accomplishment of endodontic treatment depends entirely on the awareness, good knowledge, and skill of the clinician. The foremost objectives of successful endodontic treatment are highly dependent on the identification of all root canals, accessibility, and thorough cleaning and shaping of the root canal system [2]. In some cases, the canals may be difficult to locate due to morphological variations or internal calcifications. Failure to thoroughly clean and shape all the canals can lead to persistent infection, recurrent pain, incomplete healing, and eventual treatment failure [3-7]. Therefore, it is important to understand the anatomy of the root canal system and its variations.

The maxillary first molar is one of the most complex teeth to treat due to the variable anatomy of its root canal system [8,9]. The presence of a second mesiobuccal (MB2) canal is often overlooked or undetected in routine clinical examination, leading to missed canals and treatment failure. The presence of an MB2 canal is highly variable and ranges from 35% to 96%, as per a study of the Chinese population [10]. The use of cone beam computed tomography (CBCT) has revolutionized the diagnosis and treatment planning of complex anatomical structures in dentistry.

The diagnosis of a missed MB2 canal can be challenging and relies on a combination of clinical and radiographic examination. Clinical signs of a missed canal may include persistent pain or swelling, sinus tract, or incomplete resolution of symptoms after root canal treatment [11]. Radiographically, a missed canal may be indicated by the presence of a radiolucency or a periapical lesion [12]. However, conventional radiographs have limited ability to visualize the complex anatomy of the root canal system, and the presence of a missed canal may be undetected [13]. Therefore, the use of CBCT has become the gold standard for visualization of the root canal system and detection of a missed MB2 canal [14].

Cone-beam computed tomography (CBCT) is a three-dimensional advanced imaging technology that provides cross-sectional views of the maxillary first molar, allowing for the visualization of complex root canal anatomy [15]. The use of CBCT in the secondary assessment of missed canals has been shown to significantly increase the detection rate of MB2 canals, ranging from 50% to 89% [10,13]. It helps to enhance the accuracy of diagnosis and treatment planning, reduce the risk of procedural errors, and improve treatment outcomes. CBCT images can also minimize the need for exploratory surgery, thereby reducing the patient's discomfort and risk of complications [16,17].

Another advantage of CBCT is the ability to assess the quality of previous endodontic treatment, such as the presence of overfilled or underfilled canals, apical transportation, or perforation. These findings can guide the treatment plan for the management of the missed MB2 canal, improving the chances of successful outcomes [18,19]. Henceforth, the present study aimed at the prevalence of missed MB2 canals in endodontically treated teeth and its correlation with periapical pathology by using CBCT scans.

## Materials and methods

Study design and population

This retrospective study was conducted comprising 80 participants with a gender ratio of 1.35:1 (46 males vs. 34 females) who had undergone CBCT scans for a variety of diagnostic purposes, including implant planning, surgical extraction of impacted teeth, endodontic evaluations, and various treatment modalities, between January 2023 and June 2023 at a private CBCT center in Indore.

Inclusion and exclusion criteria

Patients with a mean age of 42.1±14.836 years (age range: 7.0-72.0 years) and with their asymptomatic endodontically treated upper maxillary first molars (no periapical lesions, resorption, or canal calcification, no cemented posts or large coronal restorations) were included in this study, whereas non-endodontically treated teeth were excluded in the current study.

Data collection and analysis

All participants signed an informed consent and their data was used anonymously for research purposes. The CBCT machine used was KODAK 9000 (CMOS sensor, continuous mode and 12-28 sec scan time, 90-500 μm voxel size, and 5x3.5 cm FOV). All images were assessed independently by an endodontist and an oral radiologist. Observers were instructed to examine the following: (1) presence/absence of MB2 canals. (2) Number of filled/unfilled MB2 canals. (3) Location of MB2 canal. (4) Periapical condition in association with MB2 canal.

In the present study, axial sections were acquired to determine whether the MB2 canal existed and where it was located. The radiographic apex of each mesiobuccal root image was scrolled axially from the canal orifice. The canal has to be traceable in successively cut sections to at least half the length of the root to be documented as a second mesiobuccal canal. Statistical analysis was performed using the SPSS version 20.0 (Chicago, IL: SPSS Inc.). Significance was set at p<0.05.

## Results

**Figure 1:**
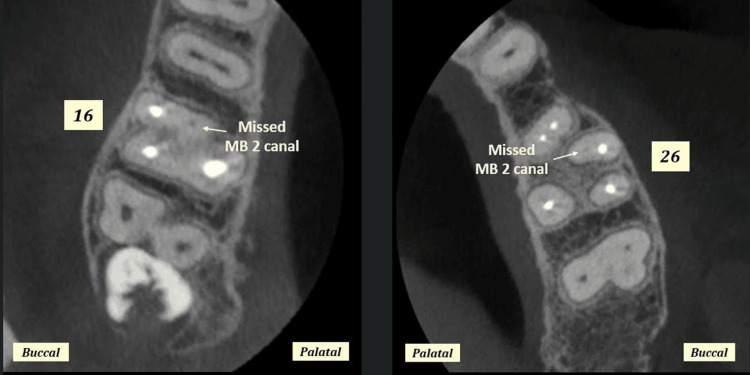
CBCT images showing missed mesiobuccal root canal configurations in axial sections of right and left maxillary first molars at cervical root third level. MB: mesiobuccal; MB2: second mesiobuccal canal; CBCT: cone beam computed tomography

**Figure 2:**
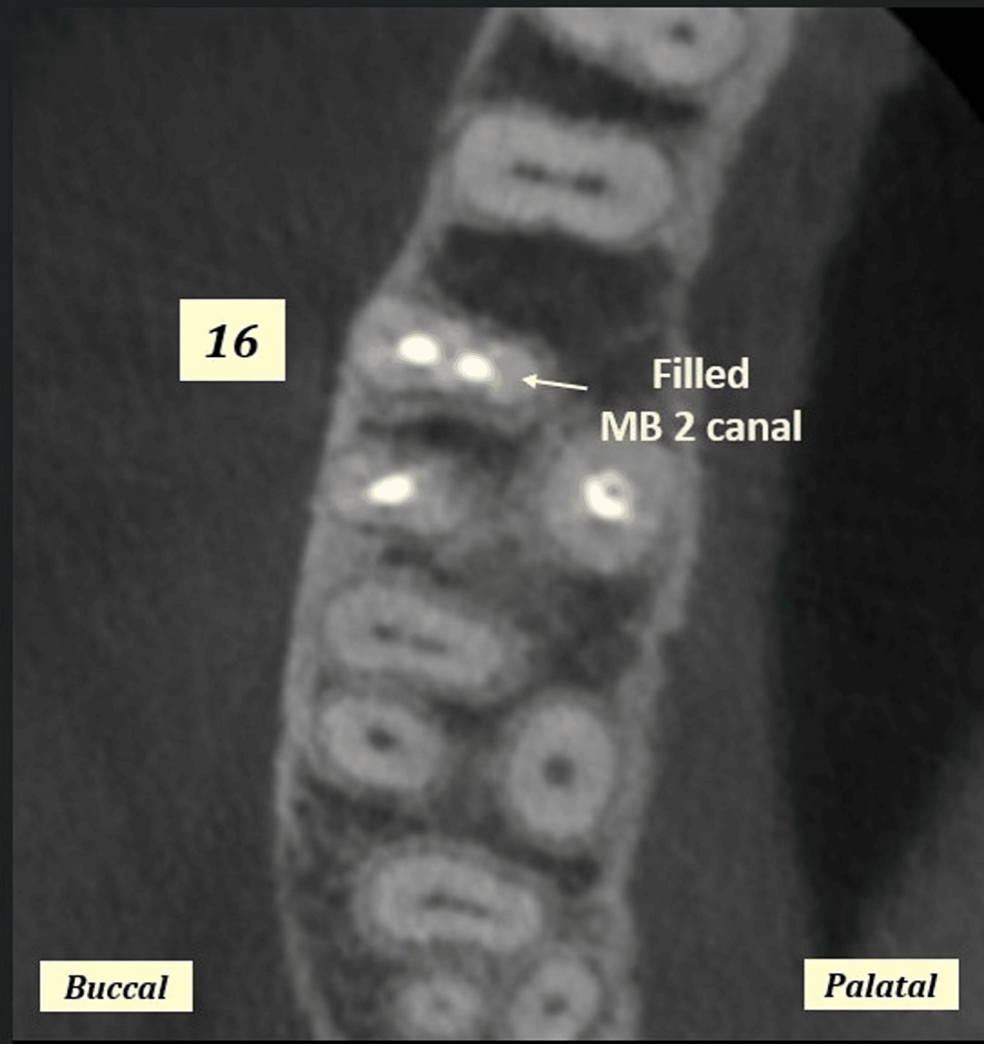
CBCT images showing filled mesiobuccal root canal configurations in axial sections of right maxillary first molars at middle root third level. MB: mesiobuccal; MB2: second mesiobuccal canal; CBCT: cone beam computed tomography

**Figure 3:**
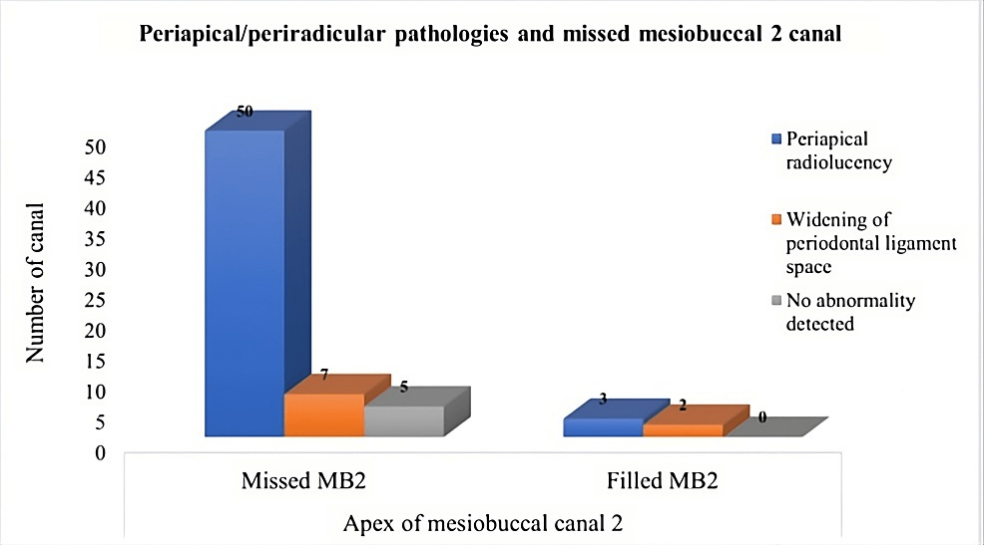
Graphical representation of the periapical/periradicular pathologies in filled and missed mesiobuccal 2 canal. Chi-square value: 3.490, degree of freedom: 2, and p-value: 0.175

The study included 80 participants with a mean age of 42.1±14.836 years (age range: 7.0-72.0 years). The male-to-female ratio was 1.35:1 (46 males vs. 34 females). The study included 39 (48.8%) right maxillary first molar and 41 (51.3%) left maxillary first molar (Figure 1). Overall, in 62 (77.5%) maxillary first molars, MB2 was missed by the practicing dentist, and in 13 (16.3%) maxillary first molars MB2 canal was not present (Tables 1, 2). Overall, the incidence of missed MB2 canal was 92.5% (62/67), and it was non-significantly different between males and females and between maxillary first molar of the right and left side (p>0.05) (Tables 1, 2). Of all the maxillary first molar with MB2 canal (n=67), 53 (79.1%) canals had periapical infection, five (7.5%) showed widening of periodontal ligament space, whereas nine (13.4%) had no abnormality (Figures 2, 3). The apex of the MB2 canal was separated into 26 (38.8%) teeth and was placed in apical third in 24 (35.8%), in middle third in 15 (22.4%), and in cervical third in two (3.0%) maxillary first molars.

**Table 1:** Frequency distribution of teeth based on findings of mesiobuccal canal. CBCT: cone beam computed tomography

CBCT finding	Male	Female	Total
Missed mesiobuccal canal 2	33 (71.7%)	27 (79.4%)	60 (75.0%)
Missed mesiobuccal canal 1 and 2	2 (4.3%)	0 (0.0%)	2 (2.5%)
Filled mesiobuccal canal 2	2 (4.3%)	3 (8.8%)	5 (6.3%)
Mesiobuccal canal 2 not present	9 (19.6%)	4 (11.8%)	13 (16.3%)
Total	46 (100.0%)	34 (100.0%)	80 (100.0%)

**Table 2:** Frequency distribution of right and left teeth based on findings of mesiobuccally canal (MB2). Chi-square value: 0.227, degree of freedom: 3, p-value: 0.973, CBCT: cone beam computed tomography

CBCT finding	Maxillary right first molar	Maxillary left first molar	Total
Missed mesiobuccal canal 2	30 (76.9%)	30 (73.2%)	60 (75.0%)
Missed mesiobuccal canal 1 and canal 2	1 (2.6%)	1 (2.4%)	2 (2.5%)
Filled mesiobuccal canal 2	2 (5.1%)	3 (7.3%)	5 (6.3%)
Mesiobuccal canal 2 not present	6 (15.4%)	7 (17.1%)	13 (16.3%)
Total	39 (100.0%)	41 (100.0%)	80 (100.0%)

## Discussion

Endodontic success is dependent on comprehensive treatment of the complete pulpal system. For diagnosing and treating dental issues, the diverse variations in root and root canal morphology, especially in multi-rooted teeth, continue to pose persistent challenges. These difficulties arise from factors such as the intricate location of the roots, their narrow dimensions, calcification, and the presence of pulp stone debris [20]. For an endodontic procedure to be successful, dentists must possess a detailed understanding of root canal configurations and their variations.

Missed or unreachable canals during therapy could have an impact and may be the cause of endodontic failure [21]. Due to the root curvatures, accessory canals, and changes in the internal anatomy of the maxillary first molars, clinicians should perform the endodontic treatment cautiously while treating them. Typically, the maxillary molar shows complexity in its mesiobuccal root with a single palatal canal that is wide mesiodistally [22,23]. It is common to locate the MB2 canals in the mesiobuccal root [23].

More than 70% of maxillary first permanent molars have an MB2 canal, according to in vitro investigations. A study conducted by Imura et al. revealed the existence of the MB2 canal at the anatomical apex in 90% of cases [22]. Similarly, research by Kulid and Peters found an MB2 canal in the coronal half of 95.2% of the roots. This additional canal was detected in 54.2% of cases using hand instruments, 31.3% using a bur, and 9.6% using a microscope [23].

Because there are no clear-cut symptoms in that range that can be directly associated with a missed canal issue, diagnosing missed canals can be problematic. It has been determined that the presence of a missed canal on an endodontically treated tooth may be considered a clinically relevant condition by looking at its prevalence and correlation with periapical pathosis [24,25].

Missed/not present MB2 canals

The present study included 39 (48.8%) right maxillary first molar and 41 (51.3%) left maxillary first molar (Figures 1, 3). Overall, in 62 (77.5%) maxillary first molars, MB2 was missed while performing the endodontic treatment and in 13 (16.3%) maxillary first molars MB2 canal was not present. Overall, the incidence of missed MB2 canal was 92.5% (62/67) (Tables 1, 2).

Filled/unfilled canals

In the present study, 6.3% were filled in first molars irrespective of gender and side, which was similar to the study done by Shetty et al. where filled MB2 canals were present in 22% and findings by Sempira and Hartwell that incidence of filled MB2 canals were less than 40% (Tables 1, 2 and Figures 1, 2) [20,26]. Nevertheless, on the contrary, the result of the present study was lower compared to that of Buhrley et al., where the findings for the microscope, dental loupes, and no magnification groups were 71.1%, 62.5%, and 17.2%, respectively [27].

Location of MB2 canal

In the present study, it was placed in apical third in 24 (35.8%), in middle third in 15 (22.4%), and in cervical third in two (3.0%) maxillary first molar, unlike a study done by Al-Habib and Howait where they reported the location of joining was 14 (23%), 17 (27%), and 31 (50%) coronal, middle, and apical third, respectively [28].

Impact of the filled/unfilled canals on periapical region

Of all the maxillary first molar with MB2 canal (n=67), 53 (79.1%) canals had a periapical infection, five (7.5%) showed widening of periodontal ligament space, whereas nine (13.4%) had no abnormality. The prevalence rate was more on comparing with the study done by Shetty et al., where unfilled MB2 canals showed periapical radiolucencies in 73% of cases, and less in a study done by Karabucak et al., where it was noted as 82.8% [20,24].

Owing to the complexity and variation in mesiobuccal roots, the clinician could miscue canals during the procedure. These unattended canals could serve as a bacterial reservoir going forward, as evidenced by the increased frequency of periapical disease [9,29].

Limitations

The first and foremost limitation is its smaller sample size. The study lacks the essential histologic or microbiological component necessary to establish a clear connection between untreated periapical pathosis and the root canal. It is advisable to conduct further research with more increase in sample size to better comprehend the relationship between histologic and microbiological diagnosis, as well as teeth with endodontic treatment but untreated periapical pathosis and canals.

## Conclusions

Because of the significant occurrence of periapical lesions linked to missing canals in the present study, untreated or unfilled root canals should be regarded as a crucial consideration for the development of any periapical pathology. Consequently, physicians should appropriately understand the anatomical structures, root canal system, and potential changes before the commencement of the endodontic procedure to minimize the potential for missing canals during treatment.
